# Progress in Otology

**Published:** 1901-09-21

**Authors:** 


### PROGRESS IN OTOLOGY.

(*Continued from page* 399.)

A correct knowledge of the course which the air takes in passing through the nose has important bearings upon aural surgery, as without it we are apt to misdirect our efforts for the relief of nasal insufficiency and the establishment of efficient ventilation of the middle ear. In other words, if we recognise the inferior meatus as the main inspiratory air-path, and the middle and upper meati as having a very subsidiary, if any, importance in the direction taken by the current, we may suffer obstructions in these regions to go untreated which all the while are creating manifest aural phenomena. The downward aspect of the anterior nases in itself suggests the course taken by the inspired air in following its comparatively straight line to the entrance of the middle meatus, yet the inferior is the so-called respiratory tract, to reach which the air must take a strong curve backwards at the limen vestibuli. Zarniko proved by experiment a considerable time ago, the upward course of the inspired air, and later observers, amongst them C. A. Parker,[6] have demonstrated that it takes a still higher one than even he believed. Chevalier Jackson[7] directs attention to the influence of certain abnormalities of the septum (usually taken little notice of) upon the inspiratory blast as it descends to reach the posterior choanæ on its way to the nasopharynx. These are the small pads or swellings often seen upon the septum posteriorly, close to its free border. By the aid of experiments on the cadaver, Jackson has endeavoured to prove that these swellings deflect the course of the atmosphere in a lateral direction on to the Eustachian cushions and tube mouths. He adduces clinical evidence in support of his contentions and reports seeing deposits of black material, over the Eustachian region in aural patients, the subjects of these growths. He records that the aural trouble disappeared when the growths were extirpated by the curette. Jackson recommends this practice in every case in which these swellings occur, as a prophylaxis against deafness and tinnitus. This advice is scarcely likely to be followed unless the growths attain dimensions sufficient to cause manifest nasal obstruction.

Remedies for chronic non-suppurative otitis media, the bane of otologists, still come, and "practical cures" are still reported. At the last Otological Congress, Turnbull passed in review the measures which he considered have stood the test of the last 35 years. Foremost amongst these the Politzer bag, after which, Delstanche's raréfacteur, and one or two vibratory methods, were almost the only ones mentioned. Perhaps the bougie and intratympanic injections might be included with these as occasionally rendering valuable aid. Yet it would be a mistake to adopt a hypercritical or cynical attitude towards new remedies enthusiastically eulogised by their advocates lest we stifle research by so doing, and postpone the day, apparently still so far distant, when this inveterate disease shall be placed on the list of curable infirmities. Unfortunately a more accurate knowledge of the natural history and pathology of chronic dry deafness is still a desideratum, and although a clear dividing line between the catarrhal and sclerotic forms cannot be drawn, a full recognition of the two distinctive types is of considerable importance in practice. Amongst the therapeutic agents recently brought forward and awaiting the test of fuller experience is thermotherapy, and there can be no questioning the honesty which Messrs. Lermoyez and Mahu[7] have exhibited in their account of failures and successes with its employment. The apparatus perfected by these authors with great ingenuity, consists of (1) an air reservoir; (2) a spiral metal tube in case with gas flame beneath, for heating the air supplied from the reservoir; (3) a conducting tube and canula for conveying the hot air to the affected parts. The temperature of the heated air ranges from 70° to 90° C., and is conveyed to the cavities of the nose under illumination through a nasal speculum by means of a special canula provided with a thermometer to indicate the temperature, and terminating in an ivory extremity. The air is directed to the required point by guiding the canula with the right hand, the nozzle being kept about three or four millimetres from the surface. Lermoyez and Mahu claim to have attained their best results in the treatment of diseases of the nose, *i.e.*, rhinorrhœa, anosmia, and others having a neurotic basis, but they enumerate the following aural affections as being highly suitable for thermo-therapy.—(1) Otalgias, secondary to affections of the pharynx and larynx or nose, or supervening upon certain congestive attacks which are sometimes the first stage of acute otitis. (2) Recent stenoses of the Eustachian tube accompanied by noises in the ear and deafness. No benefit is to be expected for tinnitus resulting from anchylosis of the stapes, or deafness due to confirmed sclerosis. An examination of the list of cured cases inclines one to ask whether the same amelioration in the hearing might not have been gained by older and simpler methods, though indirectly the ear may profit by treatment of the intractable nasal neuroses, when both co-exist. If the hot air is to be directed through the Eustachian tube, this is effected by means of the catheter. Thermo-therapy has also been employed for the treatment of chronic middle ear catarrh in America though in a different manner and quite independently of Lermoyez. This is a device by G. W. Hopkins,[8] of Cleveland, Ohio, and by it the hot air is directed from a simple oil stove along a sleeve to the side of the head, where it comes in contact with a large pad of dry gauze placed over the affected ear, narrow strips of gauze being also packed into the meatus. Hopkins mentions a case which he states has stood the test of four years, and he has treated 62 characteristic cases since with but four failures—all old people with extensive labyrinthine involvement. Hopkins believes the intense heat (which is allowed to rise to 400° Fahr.) "stimulates the circulation through the blood supply on the posterior side of the manubrium, causing absorption of the articular deposits, removing atrophy and relieving the rigidity of the tensor tympani. The ossicles receive the full benefit of the heat, and adhesions between portions of the ossicular chain and the adjoining walls can be readily removed." There is a naïve optimism about the writer's style here, and in his facile explanation of the "brilliant results" he has obtained, which makes one chary of being too sanguine as to the future of this remedy, and in the description of the one case he narrates there is a mixing together of conditions characteristic of the two distinct types which justifies some doubt in the matter of diagnosis; thus a nose showing "typical hypertrophic rhinitis" is associated in a case with ankylosis of the ossicles from sclerosis, slight dilatation of the Eustachian tube and labyrinthine involvement.

The treatment of sclerosis by purely operative means is not supported by the most modern and recently-accepted views of its pathology. Ricardo Batey, Siebermann,[9] and others have followed up Politzer's original investigations and discoveries, and unite with him in stating that relief by surgical means is hopeless. The part most frequently and earliest affected is the wall of the labyrinths, especially the antero-superior part of the pelvis ovalis, which in the end leads to fixation of the stapes. Isolated foci also occur in the turns of the cochlea, the lumen of which may be invaded by extension of spongy bone inwards. Hence stapedectomy must be regarded as useless, since after its performance the proliferation of bony tissue effectually closes up the fenestra ovalis.

There is scarcely any practical point in aural surgery of more interest and importance than the contamination of the ears through the passage of infective material from the nasal cavities or their adnexa. Of the risk to the integrity of the ear incurred by operative interference in the nose, every rhinologist is aware; the wonder is that accidents are not more frequent. It is believed that nature closes the Eustachian tubes in the presence of danger, but if this faculty does exist it may be defective in some cases. Aural inflammation after a nasal case due to carelessness in the use of the douche or to want of cleanliness on the patient's part, invariably leads to a mastoid operation, owing to its septicity. In a case recently mentioned by MacBride,[10] the source of infection was conclusively traced to a collection of pus in the antrum of the opposite jaw. The drum membrane ruptured spontaneously, but not freely, bulged and pouted, and discharged pus. In spite of paracentesis, no improvement in the condition of the ear occurred, till the maxillary antrum was opened and drained. Grunert and Zeroni[11] report a case of lepto-meningitis following mastoiditis from the use of the nasal douche in a woman aged 57. Purulent basilar meningitis spreading to the lateral ventricles and cerebellum carried the patient off six days after the opening of the mastoid.

The great discussion on the indications for opening the mastoid[12] in chronic suppurative otitis media at the International Otological Congress in London, in which so many distinguished men participated, will long remain a landmark in the history of aural surgery, but since then there has been no standing still in the march of knowledge. It will be interesting, before passing on to notice the most recent utterances on the subject, to glance at some of the principles laid down at the London Congress by that consummate artist in surgery, Mr. MacEwen. So sound and comprehensive are they that it may well be questioned if they can ever exert a less influence over the mind of the surgeon than they assuredly do now. Here is a useful practical rule to guide an opinion on the question of operation. "When a pyogenic lesion exists in the middle ear, or its adnexa, which is either not accessible, or cannot be effectually eradicated through the external ear, the mastoid antrum and cells ought to be opened." The freedom which MacEwen reserves to himself in operating (being guided entirely by the circumstances of the case, in preference to following any prescribed rules), is exemplified in the following words:—"In opening the mastoid, he does not follow the classical operations of Küster, Stacke, or Schwartze, but proceeds first to open at the base of the supra-meatal triangle. From that point he follows the pathological lesions anteriorly into the middle ear, especially exposing and scrutinising the attic and antrum. He then passes downwards through the mastoid cells toward the sigmoid sinus, following the pyogenic erosions wherever they lead in that direction, and when necessary exposing the knee of the sigmoid sinus." MacEwen gives the three following reasons for treating persistent purulent otitis media by the mastoid operation in preference to that by way of the external auditory meatus:—(1) All the affected area is exposed to ocular inspection. (2) It is possible to secure asepsis. (3) By the formation of vascular tissue, it is possible to raise an efficient barrier against pyogenic extension to the most vulnerable parts of the brain and the sinus.

[6] Jour. of Laryngology. June, 1901. [7] Transactions of the French Society of Otology, etc.. Tome XVI., Part I. [8] Med. Rec., June 1, 1901. [9] Jour. of Laryngology. Oct. 1900. [10] Lancet, May 18, 1901. [11] Archiv. f. Ohrenk., Aug. 3, Vol. XLVI. [12] Transactions of the 6th Intern. Otol. Cong., Lond. 1900.

### PROGRESS IN GYNÆCOLOGY.

(*Continued from page* 397.)

